# High anti-Müllerian hormone (AMH) is associated with increased risks of ectopic pregnancy in women undergoing fresh embryo transfer cycle, a cohort study

**DOI:** 10.1186/s12958-022-01038-6

**Published:** 2023-02-03

**Authors:** Kai-Lun Hu, Shan Li, Sarah Hunt, Rui Yang, Huiyu Xu, Rong Li

**Affiliations:** 1 Beijing Key Laboratory of Reproductive Endocrinology and Assisted Reproductive Technology and Key Laboratory of Assisted Reproduction, Department of Obstetrics and Gynecology, Ministry of Education, Center for Reproductive Medicine, Peking University Third Hospital, Beijing, China; 2 Department of Obstetrics and Gynaecology, Monash University, Clayton, VIC 3168 Australia; 3 Department of Obstetrics and Gynecology, Peking University Third Hospital, No. 49 HuaYuan North Road, HaiDian District, Beijing, 100191 People’s Republic of China

**Keywords:** Anti-Müllerian hormone, Fresh embryo transfer, Ectopic pregnancy, Intrauterine pregnancy, IVF

## Abstract

**Background:**

Ectopic pregnancy is more common amongst assisted reproduction cycles and is a cause of significant maternal morbidity. Few predictive markers exist to help identify and modify risk of ectopic pregnancy in preparing for embryo transfer. The relationship between serum and AMH and ectopic pregnancy rate is unknown.

**Methods:**

This was a retrospective cohort study investigating women who underwent fresh embryo transfer cycles from January 2017 to December 2019 in Peking University Third Hospital. The primary outcome was ectopic pregnancy. Restricted cubic splines with four knots for AMH concentration (0-3, 3-6, 6-12, 12-max) were used to map out the non-linear relationship between the predicted ectopic pregnancy rate and the serum AMH concentration. Log binomial regression was used to test the crude risk ratio (cRR) and the adjusted risk ratio (aRR) after adjustment for confounders with 95% confidence intervals (CI) to determine the difference across various groups.

**Results:**

A total of 13,718 cycles in women undergoing fresh embryo transfer were eligible for analysis. The ectopic pregnancy rate was 1.3% per embryo transfer cycle initiated and 3.3% per pregnancy. Serum AMH concentrations were higher amongst women with ectopic pregnancy than in women with a confirmed intrauterine pregnancy or heterotopic pregnancy or who did not become pregnant (Mean levels: 4.0 ng/ml vs 3.2 ng/ml, 1.7 ng/ml, and 2.8 ng/ml). An AMH concentration of 7 ng/ml represented the best cut-off value to predict ectopic pregnancy. The ectopic pregnancy rate was 3.4% per cycle and 7.5% per pregnancy in women with AMH levels ≥ 7 ng/ml; and 1.2% per cycle and 2.9% per pregnancy in women with AMH levels < 7 ng/ml. Serum AMH concentration ≥ 7 ng/ml was associated with an increased risk of ectopic pregnancy in all fresh embryo transfer cycles started (aRR = 2.35 (1.45, 3.58)) as well in women who became pregnant (aRR = 2.23 (1.49, 3.33).

**Conclusions:**

Baseline AMH concentration ≥ 7 ng/ml is associated with an increased risk of ectopic pregnancy in fresh embryo transfer cycles.

**Supplementary Information:**

The online version contains supplementary material available at 10.1186/s12958-022-01038-6.

## Background

Assisted reproductive technology (ART) has assisted millions of couples with subfertility since its advent in 1978 [1, 2]. However, the risk of adverse outcomes, such as ectopic pregnancy, placenta previa, preterm birth, low birth weight, et.al. are significantly increased in ART pregnancies [3, 4].

Ectopic pregnancy is a life-threatening complication of early pregnancy characterized by embryo implantation outside the uterus [5]. A good understanding of the factors that increase the risk of ectopic pregnancy is essential to improving pregnancy outcomes as well as preserving future fertility [6]. Accumulating evidence suggests that ART is an independent risk factor for ectopic pregnancy, despite the embryo being transferred directly into the uterus [7, 8]. The rate of ectopic pregnancy varies from 1.4 - 3.5% in ART pregnancies, with variation across countries [7–11]. Risk factors for ectopic pregnancy in women undergoing in vitro fertilization (IVF) include: tubal factor infertility, excessive ovarian response, previous miscarriage or ectopic pregnancy and thin endometrium [11–13]. Controlled ovarian hyperstimulation in IVF is associated with an increased risk of ectopic pregnancy as compared with women undergoing natural cycles [10, 12, 14]. Furthermore, the rate of ectopic pregnancy in frozen embryo transfer cycles is much lower than in fresh embryo transfer cycles [9, 15–17], suggesting that the altered tubal-uterine environment caused by the ovarian stimulation contributes to abnormal implantation of the transferred embryo.

The relation between baseline biomarkers and ectopic risk are poorly described. Serum AMH is derived from pre-antral and small antral follicles in the ovary and thus serum AMH is proportional to the size of the follicular pool [18]. Recent studies suggest an association between serum AMH levels > 7 ng/ml and decreased live birth rates in fresh transfer cycles [19, 20], indicating that high AMH levels may be associated with adverse pregnancy outcomes. Previous studies suggest that increased oocyte yield was associated with a significantly increased rate of ectopic pregnancy [21, 22]. Given that serum AMH concentration is considered the gold-standard biomarker to predict oocyte yield from ovarian stimulation [23, 24], we hypothesized that serum AMH concentration might represent a better predictor for ectopic pregnancy when compared to oocyte yield. This study aimed to investigate whether higher serum AMH concentration were associated with an increase in ectopic pregnancy rates in fresh embryo transfer cycles.

## Methods

This retrospective cohort study was approved by the Ethics Committee of Peking University Third Hospital. A total of 35,617 women who underwent their first IVF cycles that resulted in fresh embryo transfer in Peking University Third Hospital from January 2017 to December 2019 were eligible for this study. Freeze- all cycles, donor oocyte cycles, pre-implantation genetic test (PGT) cycles and cycles where measurement of AMH was omitted or no embryo was available for transfer were excluded for analysis. We also excluded cycles where the stage of embryo development at transfer and the number of embryos transferred could not be determined.

The IVF protocol in our center has been described in previous studies [19]. Briefly, follicular stimulating hormone (FSH) (Gonal-F; Serono, Geneva, Switzerland) and/or human menopausal gonadotrophin (HMG) (Pergonal; Serono) was used for ovarian stimulation in conjunction gonadotrophin-releasing hormone (GnRH) agonist (GnRH-a, Decapeptyl; Ferring, Lausanne, Switzerland) or GnRH antagonist (Centrotide; Serono) to prevent ovulation. A dose of 5000-10,000 U of human chorionic gonadotrophin (HCG, Livzon, China) was administered when two or more follicles reached 18-mm mean diameter. Oocytes were transvaginally collected under ultrasound guidance 36-38 h after HCG administration. Conventional IVF or intracytoplasmic sperm injection (ICSI) was used for fertilization and up to two embryos were transferred at the cleavage stage or blastocyst stage. Thin endometrium was diagnosed where the endometrial thickness was at or less than 7 mm on the day of trigger. Embryo quality was assessed by a well-trained embryologist according to previously described criteria [25].

All women had a baseline serum AMH measurement by an ultrasensitive two-site ELISA (AnshLabs, Webster, TX, USA) as previously described [24]. All AMH measurements were taken within 6 months of commencing a treatment cycle. Serum estradiol concentration was measured on the day of trigger (Siemens Immulite 2000 immunoassay system, Siemens Healthcare Diagnostics, Shanghai, P. R. China). The inter-assay and intra-assay coefficients of variation were less than 8% and less than 10% for AMH and estradiol concentrations respectively [24].

Ectopic pregnancy was defined as the implantation of an embryo at any site other within the endometrium and was diagnosed on transvaginal ultrasound scan or at laparoscopy. Intrauterine pregnancy was defined by the presence of gestational sac(s) on ultrasound at around 7 weeks gestation with the detection of heartbeat activity within the uterine. Heterotopic pregnancy was diagnosed when at least one embryo was found to have implanted in the uterine cavity and at least one embryos was simultanesouly implanted outside of the uterine cavity. Clinical pregnancy included ectopic pregnancy, heterotopic pregnancy, and intrauterine pregnancy in this study.

An ANOVA test or Chi-square test was used to compare variables between groups as appropriate. Log binomial regression was used to test the crude risk ratio (cRR) and the adjusted risk ratio (aRR) after adjustment for confounders with 95% confidence intervals (CI). The linear relationship between AMH concentration and the risk of ectopic pregnancy was also analyzed using log binomial regression. Directed Acyclic Graphs (DAGs) were used to identify potential confounders in the multivariable analysis [26–28] (Supplemental Fig. 1). Restricted cubic splines with four knots for AMH levels (0-3, 3-6, 6-12, 12-max) were used to map out the non-linear relationship between the predicted ectopic pregnancy rate and the serum AMH level as a continuous variable before and after adjustment for covariates [29]. We then stratified women into groups by AMH level to further confirm the results from the restricted cubic splines. Stratification and the restricted cubic splines were used to determine the best cut-off value for AMH concentration to predict ectopic pregnancy. Female age, BMI, primary infertility, parity, ectopic history, infertility diagnosis (tubal factor, ovulatory dysfunction) were considered to be confounders and were adjusted in Model 1. In Model 2, gonadotrophin (Gn) dose, number of retrieved oocytes, peak serum estradiol concentraction, endometrial thickness, cycle type (antagonist), fertilization with intracytoplasmic sperm injection (ICSI), double embryo transfer, blastocyst transfer and the availability of at least one poor-quality embryo for transfer were added to the list of cavariates in Model 1 to further detect potential risk factors for ectopic pregnancy. We first analyzed the association of AMH and ectopic pregnancy in women undergoing a first embryo transfer cycle. We then examined the association between AMH and ectopic pregnancy in women who achieved a clinical pregnancy. Heterotopic pregnancy was excluded in the primary analysis but included in sensitivity analysis. All statistical analysis was performed using Stata version 15.1 (StataCorp LLC, Texas, USA). A *p* value < 0.05 was considered to represent a statistically significant difference.

## Results

### Baseline characteristics of women in fresh embryo transfer cycles

A total of 13,718 fresh embryo transfer cycles were eligible for analysis. Of these, 8144 did not result in pregnancy, 5378 resulted in an intrauterine pregnancy, 182 resulted in an ectopic pregnancy and 14 heterotopic pregnancies were diagnosed (Supplemental Fig. 2). The ectopic pregnancy rate was 1.3% per embryo transfer cycle and 3.3% per pregnancy.

**Fig. 1:**
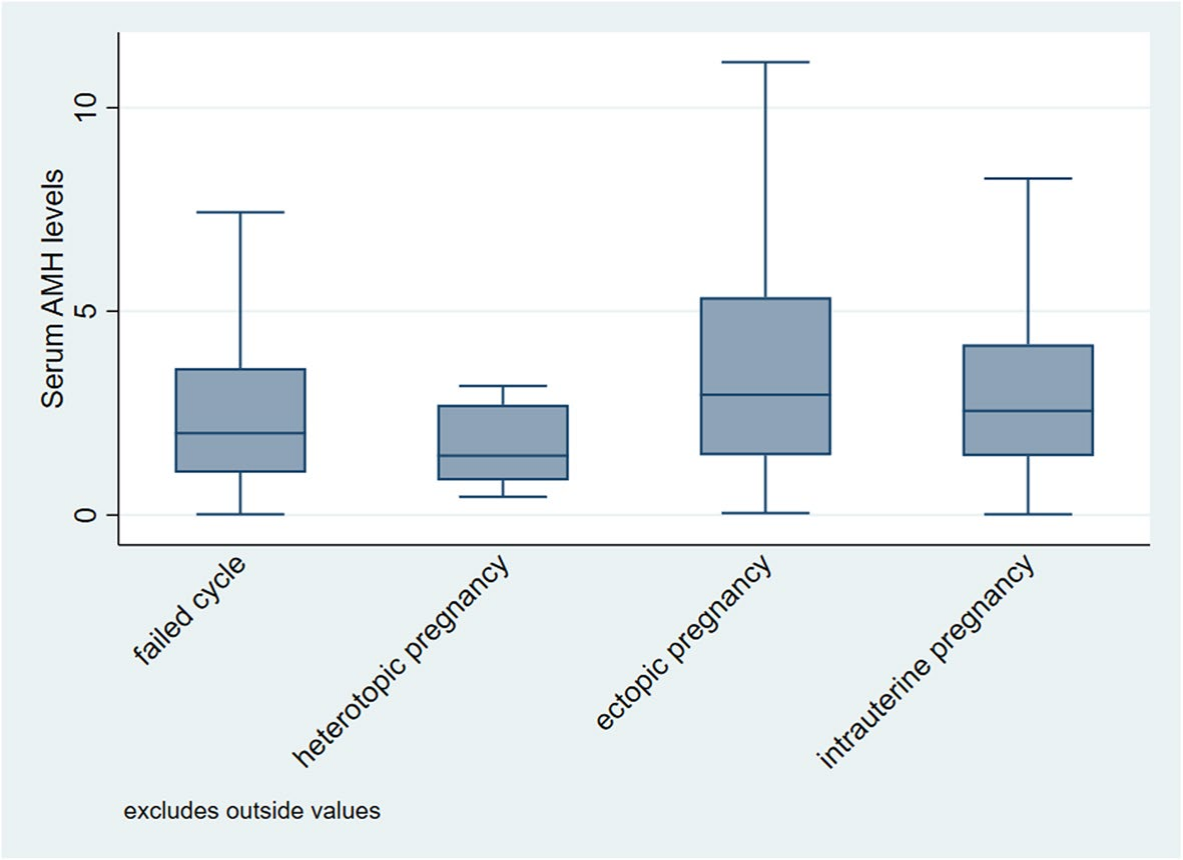
Box plot showing serum AMH levels in women with no pregnancy, ectopic pregnancy, clinical pregnancy, and heterotopic pregnancy

Women who had an ectopic pregnancy were more likely to have a previous ectopic pregnancy or a history of ovulatory dysfunction when compared with women who had an intrauterine pregnancy (14% vs 9, 18% vs 10%). They were also less likely to have a primary infertility diagnosis (49% ectopic pregnancy vs 57% intrauterine). Ectopic pregnancy occurred more frequently in GnRH agonist cycles (70% vs 50%). Women who resulted in an ectopic pregnancy had a thinner endometrial thickness and a higher rate of thin endometrium (10.1 mm vs 11.1 mm, 4% vs 1%). A diagnosis of male factor infertility and ICSI fertilization cycles were less common amongst women with an ectopic pregnancy (43% vs 51, 23% vs 33%). The total Gn dose and days of stimulation were lower in women with an ectopic pregnancy (2556 IU vs 2773 IU, 10.9 days vs 11.4 days). Male and female age, BMI, duration of infertility, number of retrieved oocytes, peak serum estradiol concentration, parity, endometriosis or tubal factor infertility diagnoses, donor sperm cycles, blastocyst transfer, double embryo transfer and the availability of at least one poor-quality embryo for transfer were not significantly different between women with intrauterine pregnancy and women with ectopic pregnancy (Table 1). The mean serum AMH concentration was higher in women with transfer cycles resulting in ectopic pregnancy than in women with no resulting pregnancy, intrauterine pregnancy or heterotopic pregnancy (4.0 ng/ml vs 2.8 ng/ml, 3.2 ng/ml, and 1.7 ng/ml) (Fig. 1).

**Table 1:** Characteristics of women undergoing the first fresh embryo transfer cycle

Pregnancy outcomes	No pregnancy	Ectopic pregnancy	Intrauterine pregnancy	Heterotopic pregnancy	*P* valuea	*P* value^b^
Number of cases	*N* = 8144	*N* = 182	*N* = 5378	*N* = 14		
Female age y^a^	33.4 (5.1)	31.8 (3.8)	31.7 (4.1)	31.8 (4.7)	< 0.001	0.93
Male age y^a^	34.8 (6.1)	32.5 (4.5)	33.1 (5.2)	35.1 (5.8)	< 0.001	0.09
BMI kg/m^2a^	23.0 (3.6)	23.1 (3.8)	22.7 (3.5)	23.7 (3.0)	< 0.001	0.09
Infertile age y^a^	3.0 (2.0-5.0)	3.0 (2.0-4.0)	3.0 (1.0-4.0)	3.5 (2.0-5.0)	< 0.001	0.99
Ectopic history	596 (7%)	26 (14%)	477 (9%)	3 (21%)	< 0.001	0.01
Reason for IVF						
Ovulatory dysfunction	898 (11%)	33 (18%)	520 (10%)	1 (7%)	< 0.001	< 0.001
Endometriosis	372 (5%)	7 (4%)	264 (5%)	2 (14%)	0.26	0.51
Tubal	5054 (62%)	98 (54%)	3232 (60%)	6 (43%)	0.01	0.09
Male	3989 (49%)	78 (43%)	2766 (51%)	7 (50%)	0.01	0.02
Primary infertility	4502 (55%)	90 (49%)	3085 (57%)	5 (36%)	0.01	0.03
Parity	771 (9%)	10 (5%)	389 (7%)	1 (7%)	< 0.001	0.37
GnRH antagonist	4947 (61%)	127 (70%)	2706 (50%)	5 (36%)	< 0.001	< 0.001
Gn days^a^	11.1 (2.5)	10.9 (2.5)	11.4 (2.5)	12.1 (3.6)	< 0.001	< 0.01
Gn doses IU^a^	2921 (1209)	2556 (1021)	2773 (1154)	3079 (970)	< 0.001	0.01
Sperm donor	180 (2%)	6 (3%)	182 (3%)	0 (0%)	< 0.001	0.95
EMT mm^a^	10.6 (1.7)	10.1 (1.5)	11.1 (1.6)	10.5 (1.6)	< 0.001	< 0.001
Thin endometrium	211 (3)%	7 (4%)	47 (1%)	0 (0%)	< 0.001	< 0.001
Oocyte yield ^a^	9 (5)	11 (4)	11 (5)	10 (4)	< 0.001	0.89
Estradiol level^a^	5609 (3847-8418)	6259 (4714-9602)	6072 (4214-9170)	5521 (3487-9012)	< 0.001	0.24
ICSI	2619 (32%)	42 (23%)	1772 (33%)	5 (36%)	0.04	< 0.01
Blastocyst transfer	508 (6%)	8 (4%)	236 (4%)	0 (0%)	< 0.001	1.00
Single embryo transfer	1532 (19%)	19 (10%)	534 (10%)	0 (0%)	< 0.001	0.82
At least one poor-quality embryo transferred	401 (5%)	1 (1%)	140 (3%)	1 (7%)	< 0.001	0.08

Data expressed with Cases (Percent), Mean (SD) or Median (IQR) as appropriate

*Abbreviations*: *EMT* Endometrium thickness, *IVF* in vitro fertilization, *ICSI* intracytoplasmic sperm injection

a. Comparison of three groups. Chi-square test or ANOVA test (marked with^a^) as appropriate

b. Comparison between intrauterine pregnancy and ectopic pregnancy

### Association of serum AMH levels and ectopic pregnancy

By using restricted cubic splines to map out the non-linear relationship between the predicted ectopic pregnancy rate and the serum AMH level as a continuous variable before and after adjustment for covariates, we found a “linear” relationship between serum AMH levels and predicted ectopic pregnancy rate per transfer cycle and predicted ectopic pregnancy rate per clinical pregnancy (Supplemental Fig. 3). The association remained in the sensitivity analysis where the heterotopic pregnancy was considered as the ectopic pregnancy (Supplemental Fig. 3).

**Fig. 2:**
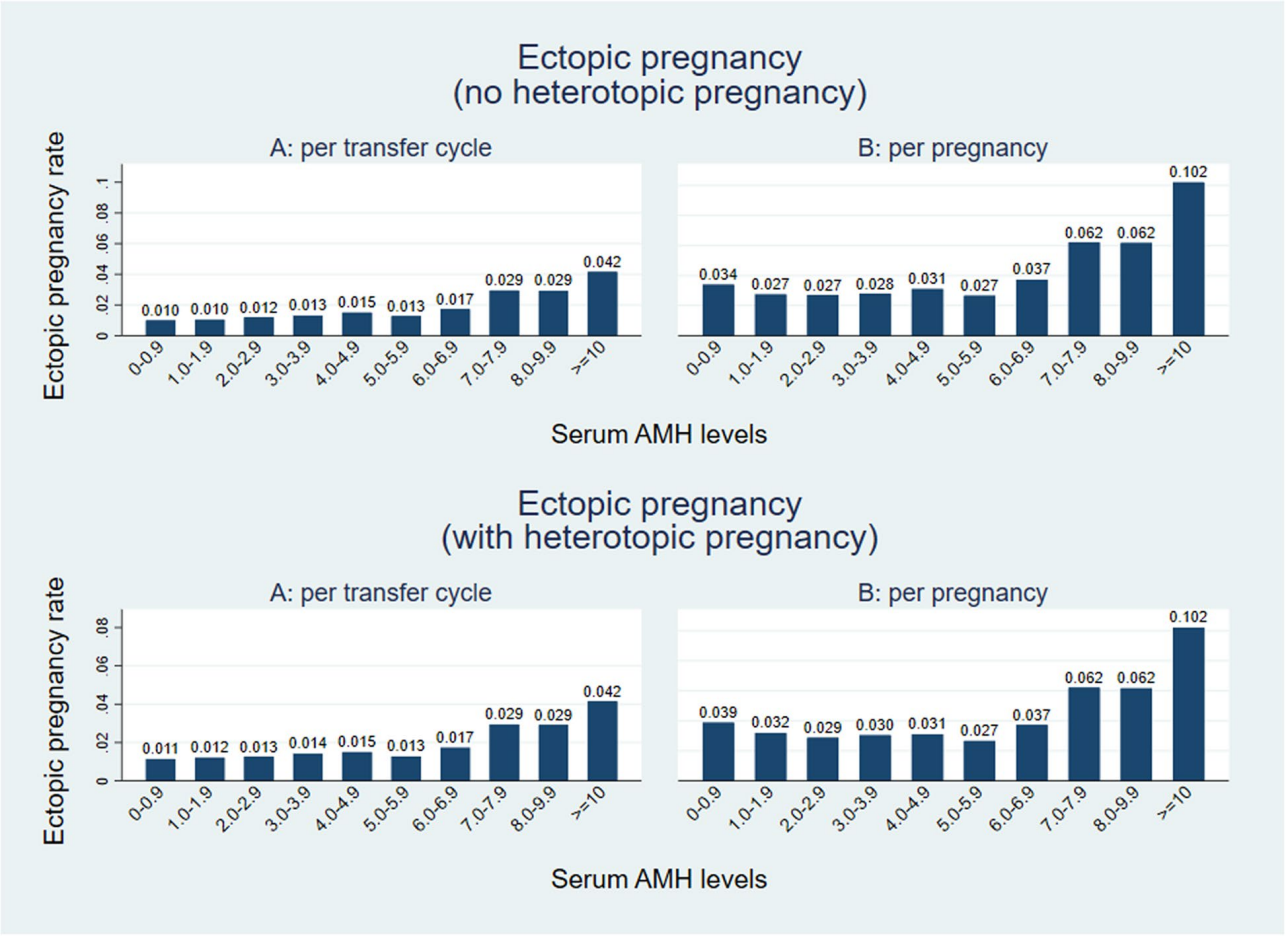
Ectopic pregnancy rate in women with different AMH levels. Left: women who underwent fresh embryo transfer cycle; Right: women who resulted in clinical pregnancy. Upper: heterotopic pregnancy was excluded; Lower: heterotopic pregnancy was included as ectopic pregnancy

We then stratified women into ten groups according to serum AMH levels (0-0.9 ng/ml; 1.0-1.9 ng/ml; 2.0-2.9 ng/ml; 3.0-3.9 ng/ml; 4.0-4.9 ng/ml; 5.0-5.9 ng/ml; 6.0-6.9 ng/ml; 7.0-7.9 ng/ml; 8.0-9.9 ng/ml; > = 10.0 ng/ml). Ectopic pregnancy rate was increased in women with AMH levels higher than 7 ng/ml (7.0-7.9 ng/ml; 8.0-9.9 ng/ml; > = 10.0 ng/ml) (2.9, 2.9, 4.2% per cycle and 6.2, 6.2, 10.2% per pregnancy, respectively) (Fig. 2). Serum AMH concentration greater than 7 ng/ml (7.0-7.9 ng/ml; 8.0-9.9 ng/ml; > = 10.0 ng/ml) was associated with an increased rate of ectopic pregnancy as compared to serum AMH levels at the range of 0-0.9 ng/ml (aRR = 2.39 (1.09-5.21), aRR = 2.43 (1.12-5.28), aRR = 3.16 (1.56-6.42) in embryo transfer cycles; aRR = 1.67 (0.78-3.57), aRR = 1.71 (0.80-3.66), aRR = 2.54 (1.28-5.03)) in clinical pregnancies, Table 2). The results were further confirmed in the sensitivity analysis (Supplemental Table 1). Thus, AMH concentration of 7 ng/ml was considered as the best cut-off value to predict ectopic pregnancy. The ectopic pregnancy rate per transfer cycle in women with baseline AMH concentration ≥ 7 ng/ml and < 7 ng/ml was 7.5 and 3.4% per clinical pregnancy and 2.9 and 1.2% per started cycle, respectively.

**Table 2:** Analysis of the association between different AMH stratifications and ectopic pregnancy (excluding heterotopic pregnancy) in women who underwent fresh embryo transfer as well as women who resulted in clinical pregnancy

AMH stratifications	Per transfer cycle			Per pregnancy		
	N	cRR (95%CI)	aRR (95%CI)	N	cRR (95%CI)	aRR (95%CI)
0-0.9 ng/ml	2723	Reference	Reference	796	Reference	Reference
1.0-1.9 ng/ml	3462	1.05 (0.64-1.72)	1.02 (0.61-1.69)	1314	0.81 (0.49-1.32)	0.85 (0.52-1.40)
2.0-2.9 ng/ml	2613	1.20 (0.72-2.00)	1.11 (0.65-1.89)	1149	0.80 (0.48-1.32)	0.83 (0.50-1.40)
3.0-3.9 ng/ml	1686	1.32 (0.75-2.30)	1.20 (0.67-2.13)	788	0.82 (0.47-1.43)	0.86 (0.49-1.51)
4.0-4.9 ng/ml	1134	1.51 (0.83-2.76)	1.26 (0.66-2.37)	548	0.91 (0.50-1.66)	0.89 (0.47-1.65)
5.0-5.9 ng/ml	706	1.29 (0.61-2.72)	1.12 (0.52-2.43)	339	0.78 (0.37-1.65)	0.79 (0.37-1.69)
6.0-6.9 ng/ml	405	1.74 (0.76-3.98)	1.46 (0.63-3.42)	188	1.10 (0.49-2.48)	1.05 (0.46-2.42)
7.0-7.9 ng/ml	306	2.91 (1.41-6.25)	2.39 (1.09-5.21)	145	1.83 (0.88-3.81)	1.67 (0.78-3.57)
8.0-9.9 ng/ml	308	2.95 (1.40-6.20)	2.43 (1.12-5.28)	146	1.82 (0.87-3.78)	1.71 (0.80-3.66)
**10-max ng/ml**	361	4.19 (2.25-7.80)	3.16 (1.56-6.42)	147	3.01 (1.64-5.52)	2.54 (1.28-5.03)

Adjusted for female age, BMI, parity, primary infertility, ectopic pregnancy history, ovulatory dysfunction, tubal factor

We also examined the association between serum AMH concentration and ectopic pregnancy in single embryo transfer cycles. Ectopic pregnancy occurred more frequently when baseline AMH was higher than 6 ng/ml (Supplemental Fig. 4) but did not reach statistical significance due to the small case number (19 in total).

### Risk factors of ectopic pregnancy

**Fig. 3:**
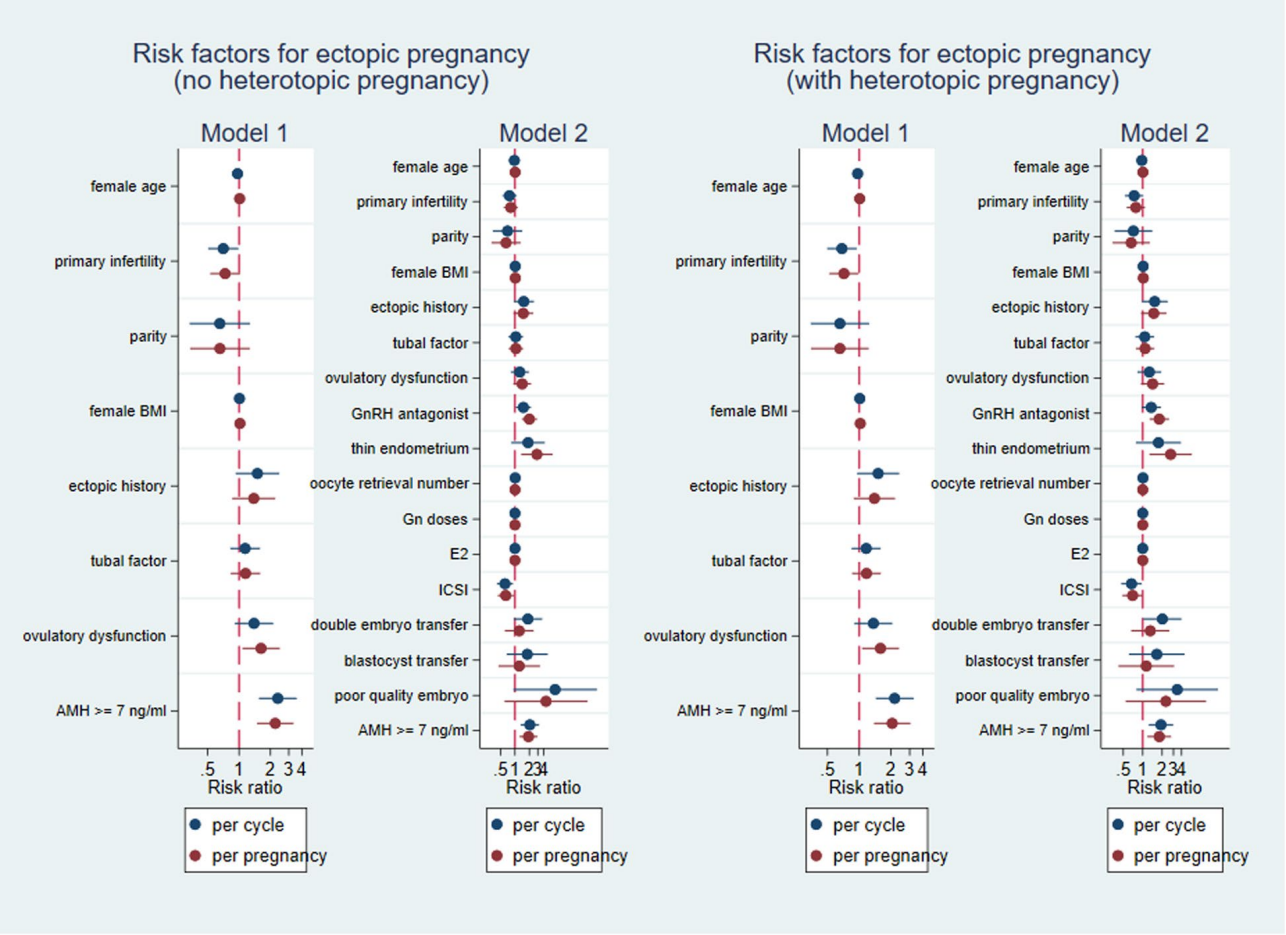
Risk factors for ectopic pregnancy in women who underwent fresh embryo transfer cycles and women who resulted in pregnancy. Left: heterotopic pregnancy was excluded; Right: heterotopic pregnancy was included as ectopic pregnancy. Model 1: female age, BMI, parity, primary infertility, ectopic pregnancy history, ovulatory dysfunction, tube factor and serum AMH levels ≥ 7 ng/ml were included in the regression analysis. Model 2: female age, BMI, parity, primary infertility, ectopic pregnancy history, ovulatory dysfunction, tube factor, serum AMH levels ≥ 7 ng/ml, Gn doses, the use of ICSI, blastocyst transfer, double embryo transfer, transfer of at least one poor quality embryo, the number of oocyte yeild, estradiol levels, thin endometrium, and the use of GnRH antagonist were included in the regression analysis

In Model 1, we demonstrated an association between serum AMH ≥ 7 ng/mL and ectopic pregnancy in fresh transfer cycles (aRR = 2.35 (1.45, 3.58)) as well as amongst clinical pregnancies (aRR = 2.23 (1.49, 3.33)) (Fig. 3, Model 1, no heterotopic pregnancy). To test whether the association was mediated by the number of collected oocytes, peak serum estradiol concentration, endometrial thickness, agonist/antagonist cycle type, Gn dose, fertilization with ICSI, embryo quality, blastocyst transfer or double embryo transfer, we added these variables in Model 2. We demonstrated that AMH concentration ≥ 7 ng/ml was still associated with an increased risk of ectopic pregnancy (transfer cycle: aRR = 2.05 (1.31, 3.20); pregnancy: aRR = 1.92 (1.25, 2.95). Figure 3, Model 2, no heterotopic pregnancy). When we included women with heterotopic pregnancy as ectopic pregnancy, the results remained in Model 1 and Model 2 (Fig. 3, with heterotopic pregnancy). The use of GnRH antagonist protocol and thin endometrium were alsorisk factors for ectopic pregnancy in women who had a clinical pregnancy (aRR = 2.00 (1.39, 2.89); aRR = 2.88 (1.35, 6.13). Figure 3, Model 2, no heterotopic pregnancy).

## Discussion

This large retrospective cohort study of 13,718 fresh embryo transfer cycles demonstrated that women with baseline AMH concentration ≥ 7 ng/ml were more likely to have an ectopic pregnancy. To the best of our knowledge, this is the first study to demonstrate an association between AMH levels and ectopic pregnancy in fresh embryo transfer cycles.

AMH is a glycoprotein secreted by the granulosa cells of the pre-antral and small antral follicles in female ovaries [18, 30]. As an ovarian reserve marker, AMH is used to predict ovarian response, the impact of chemoradiotherapy on ovarian function, and the success rate from IVF [19, 30]. To date, only one type II receptor named AMHR2 has been found for AMH in humans [31]. AMHR2 is present in several organs, including the prostate, lungs, brains, and the endometrium, suggesting that the biological effects of AMH may be much broader than initially thought [32–34]. AMHR2 is upregulated in the endometrium of women with endometriosis when compared to women without the disease [35]. Additionally, increased AMH levels in cultured human endometrial stromal cells can negatively affect cell viability and increase cell apoptosis [33, 36]. These results strongly suggest that increased AMH levels can directly affect the physiological conditions in the endometrium, which may further affect embryo implantation. However, the reason underlying the increase in ectopic pregnancy in women with high serum AMH remains unexplained. Serum AMH is positively associated with ovarian response, which may negatively affect endometrial receptivity. Accumulating evidence suggests that ovarian stimulation can alter the gene expression profile of the endometrium and negatively affect embryo implantation [37–41]. Furthermore, excessive ovarian response is an independent risk factor for ectopic pregnancy [10, 12, 22]. To summarize, both the direct and indirect effects of higher AMH concentration may contribute to the increased rate of ectopic pregnancy.

Although previous studies have suggested that oocyte yield and/or peak serum estradiol are associated with a significant increase in ectopic pregnancy [21, 22], our study did not find these markers to be significantly different between women with intrauterine pregnancy and women with ectopic pregnancy. Women with the > 20 collected oocytes and/or high peak serum estradiol concentration would routinely be converted to a freeze all cycle in our center, therefore the true impact of increase in ectopic pregnancy may be underestimated. Additionally, we found that GnRH antagonist protocol and endometrial thickness were associated with higher ectopic pregnancy rates (Fig. 3). The association between ectopic pregnancy risk and thin endometrium has been previously described [42]. Recent studies suggest that serum AMH level > 7 ng/ml in a fresh transfer cycles is associated with a decrease in live birth [19, 20], therefore the relationship between AMH and adverse pregnancy outcome requires further exploration. Consistent with these studies, we demonstrated that serum AMH ≥ 7 ng/ml is associated with a significant increase in ectopic pregnancy rate, which reaches 10% per pregnancy when AMH is ≥ 10 ng/ml. Given that ectopic pregnancy poses a substantial risk of morbidity and mortality to a pregnant woman, consideration should be given to the relative risks and benefits of a freeze all cycle in these women.

The study is strengthened by the large cohort size and the 100% follow-up. Because all data came from a single center, the clinical and laboratory practices did not substantially change over the course of the study. We analysed the effect of AMH per embryo transfer cycle as well as per clinical pregnancy, which may help apply these findings to clinical decision making. We used very “small” stratifications and restricted cubic splines to determine the best cutoff of AMH concentration for ectopic pregnancy. Furthermore, we conducted the sensitivity analysis to rule out the effect from heterotopic pregnancies. Our study has several limitations. Due to the retrospective design, residual confounding factors may be neglected, such as smoking history or social-economic factors. Our dataset did not allow us to analyze other identified risk factors for ectopic pregnancy, such as pelvic infection, systematic infection, oviduct damage, volume of transfer fluid and transfer depth [8]. The results in our study should be confirmed by future prospective cohort studies. Our study found an ectopic pregnancy rate of 2.9% per pregnancy in women with baseline serum AMH levels < 7 ng/ml. Women with AMH levels < 7 ng/ml represent 92% of total pregnant women. To demonstrate a 3% higher (5.9%) ectopic pregnancy rate per pregnancy in women with AMH levels ≥ 7 ng/ml, recruitement of 348 pregnant women with AMH ≥ 7 ng/ml and 3993 pregnant women with AMH < 7 ng/ml would be required (with a power of 80% and alpha-error of 5%).

## Conclusions

Baseline serum AMH concentration ≥ 7 ng/ml is associated with an increased risk of ectopic pregnancy in fresh embryo transfer cycles.

## Supplementary Information

**Additional file 1: Fig. S1.** DAGs was used to identify potential confounders in the multivariable analysis.

**Additional file 2: Fig. S2.** Flow chart.

**Additional file 3: Fig. S3.** Predicted probability for ectopic pregnancy in women who underwent fresh embryo transfer cycles and women who resulted in clinical pregnancy against serum AMH levels.

**Additional file 4: Supplemental Table 1.** Analysis of the association between different AMH stratifications and ectopic pregnancy (including heterotopic pregnancy) in women who underwent fresh embryo transfer as well as women who resulted in clinical pregnancy.

**Additional file 5: Fig. S4.** Ectopic pregnancy rate in women who underwent single embryo transfer.

## Data Availability

All data generated or analysed during this study are included in this published article.

## Abbreviations

hCG: human chorionic gonadotropin
rLH: recombinant LH
GnRH: ovarian Gonadotrophin-Releasing Hormone
FSH: follicle-stimulating hormone
LH: luteinizing hormone
ART: assisted reproductive technology
AMH: anti-Müllerian hormone
CI: confidence intervals
aRR: adjusted risk ratio
cRR: crude risk ratio
IVF: in vitro fertilization
PGT: pre-implantation genetic test
HMG: human menopausal gonadotrophin
ICSI: intracytoplasmic sperm injection
DAGs: Directed Acyclic Graphs
