# Supplementary figures and images for: High anti-Müllerian hormone (AMH) is associated with increased risks of ectopic pregnancy in women undergoing fresh embryo transfer cycle, a cohort study
Source: Reprod Biol Endocrinol. 2023 Feb 3;21:18. doi: 10.1186/s12958-022-01038-6 (PMC9896741; doi:10.1186/s12958-022-01038-6)

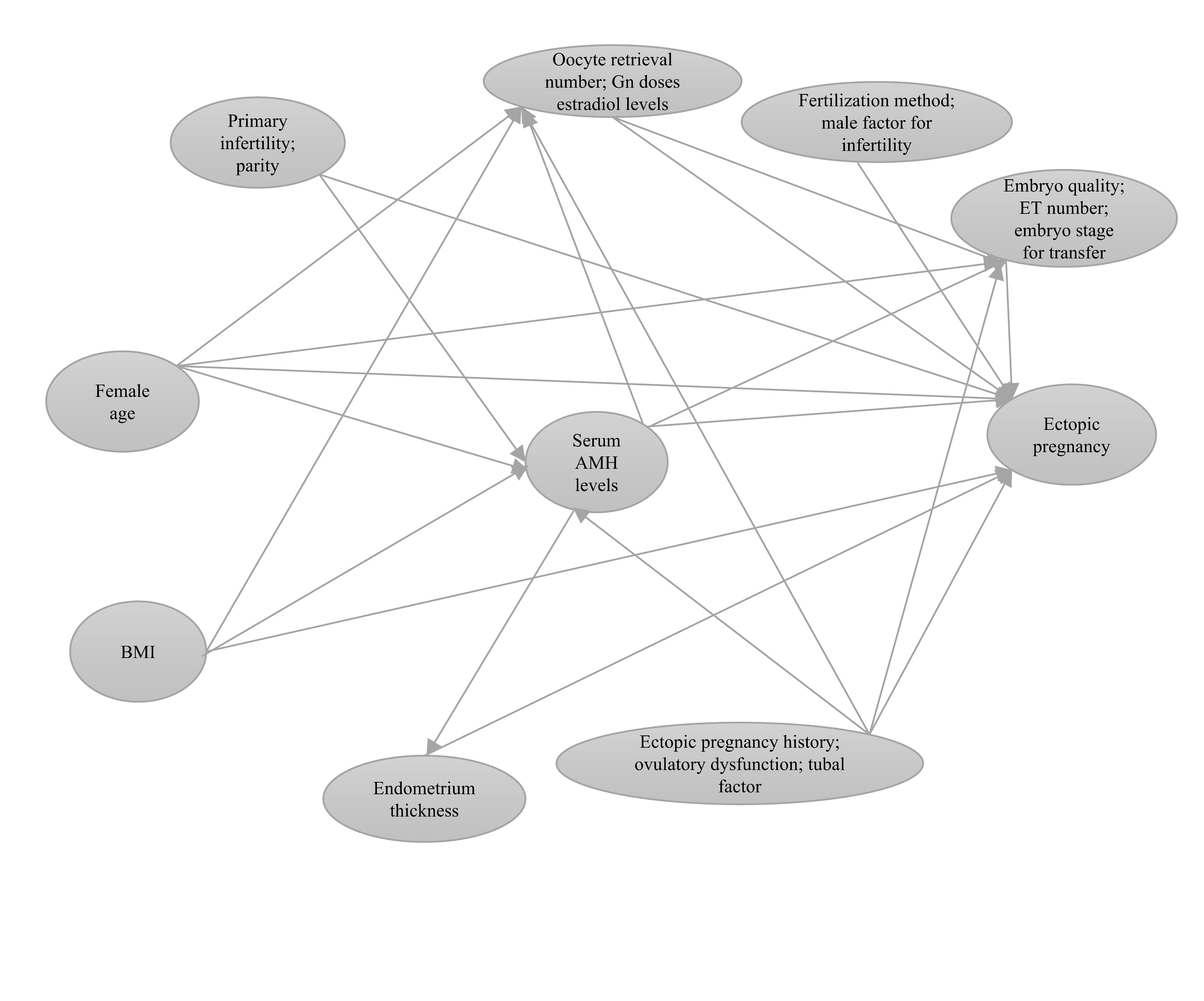

Supplement: Supplementary file 1 — Additional file 1: Fig. S1. DAGs was used to identify potential confounders in the multivariable analysis. [file 12958_2022_1038_MOESM1_ESM.tiff]

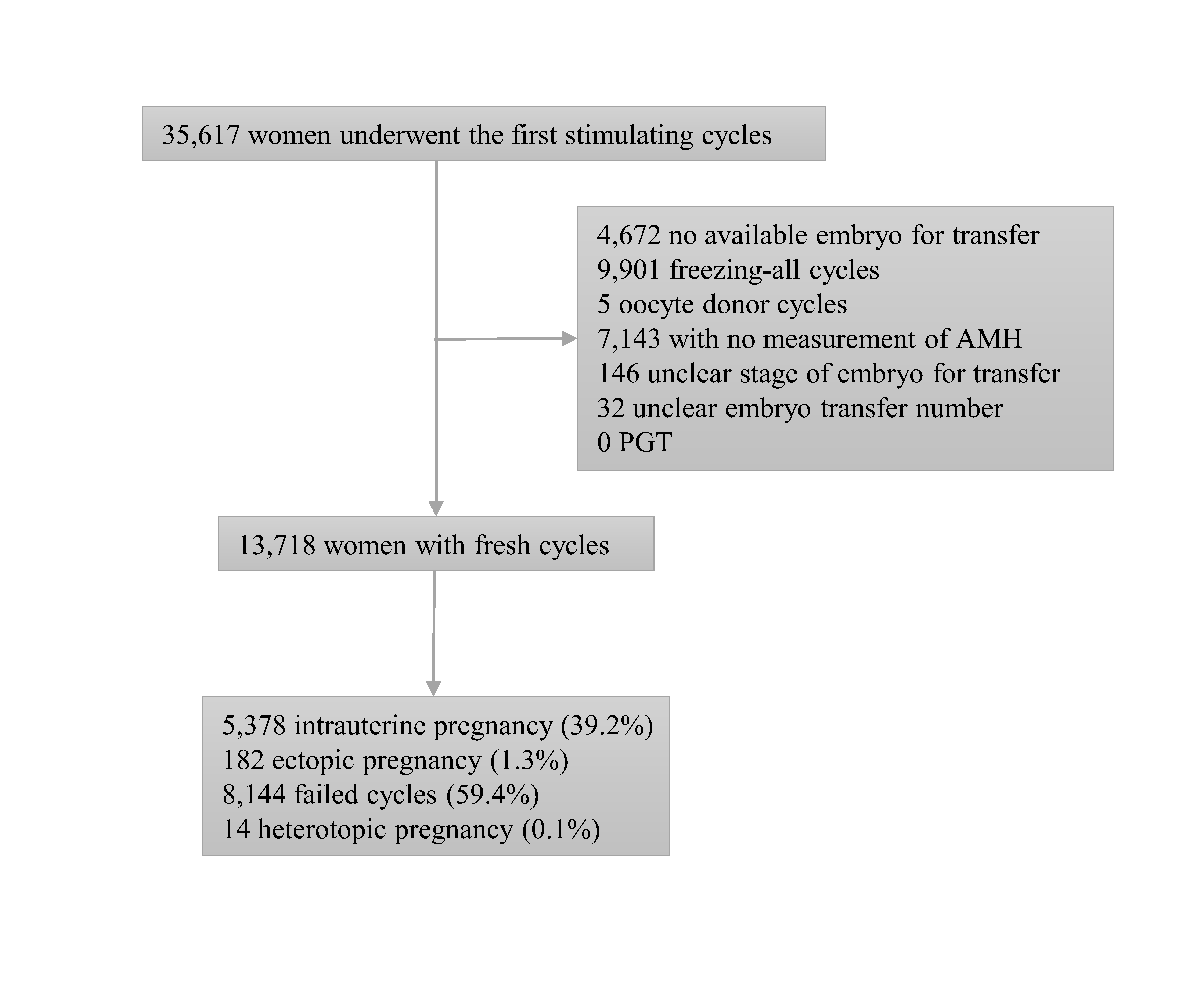

Supplement: Supplementary file 2 — Additional file 2: Fig. S2. Flow chart. [file 12958_2022_1038_MOESM2_ESM.tiff]

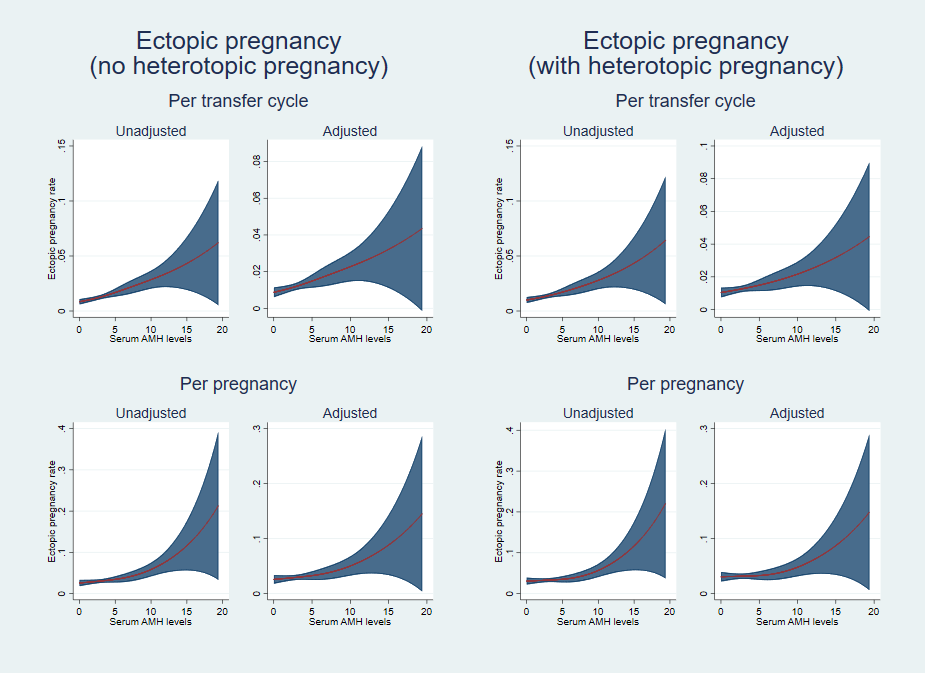

Supplement: Supplementary file 3 — Additional file 3: Fig. S3. Predicted probability for ectopic pregnancy in women who underwent fresh embryo transfer cycles and women who resulted in clinical pregnancy against serum AMH levels. [file 12958_2022_1038_MOESM3_ESM.tif]

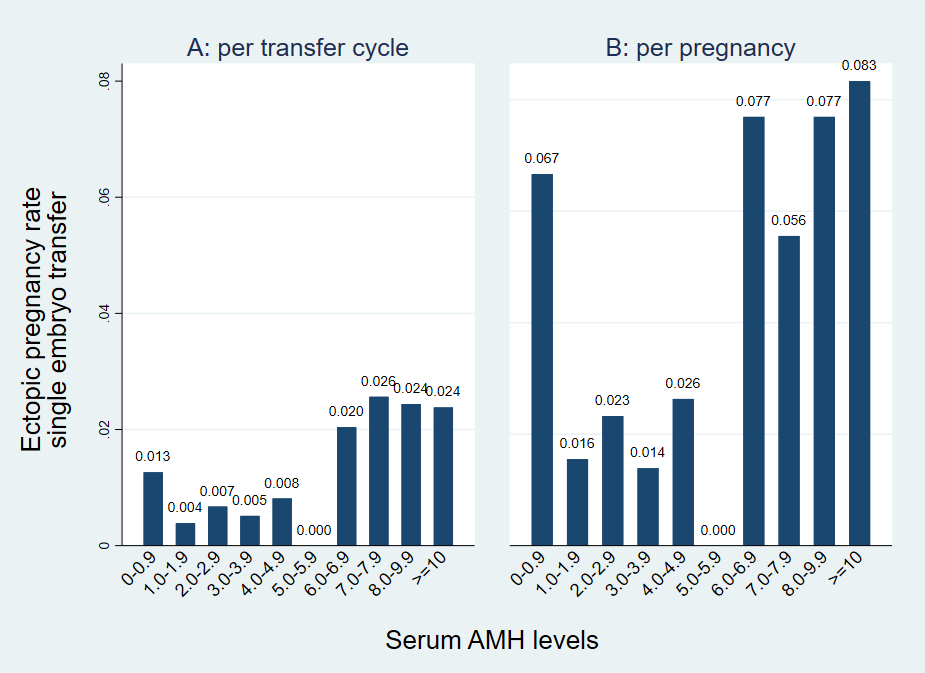

Supplement: Supplementary file 5 — Additional file 5: Fig. S4. Ectopic pregnancy rate in women who underwent single embryo transfer. [file 12958_2022_1038_MOESM5_ESM.tif]
