# Supplementary material for: High anti-Müllerian hormone (AMH) is associated with increased risks of ectopic pregnancy in women undergoing fresh embryo transfer cycle, a cohort study
Source: Reprod Biol Endocrinol. 2023 Feb 3;21:18. doi: 10.1186/s12958-022-01038-6 (PMC9896741; doi:10.1186/s12958-022-01038-6)
Supplement: Supplementary file 4 — Additional file 4: Supplemental Table 1. Analysis of the association between different AMH stratifications and ectopic pregnancy (including heterotopic pregnancy) in women who underwent fresh embryo transfer as well as women who resulted in clinical pregnancy. [file 12958_2022_1038_MOESM4_ESM.docx]

Supplemental Table 1. Analysis of the association between different AMH stratifications and ectopic pregnancy (including heterotopic pregnancy) in women who underwent fresh embryo transfer as well as women who resulted in clinical pregnancy.

| AMH stratifications | Per transfer cycle | | | Per pregnancy | | |
| --- | --- | --- | --- | --- | --- | --- |
|  | N | cRR (95%CI) | aRR (95%CI) | N | cRR (95%CI) | aRR (95%CI) |
| 0-0.9 ng/ml | 2727 | Reference | Reference | 800 | Reference | Reference |
| 1.0-1.9 ng/ml | 3468 | 1.07 (0.67-1.69) | 1.02 (0.64-1.63) | 1320 | 0.82 (0.52-1.30) | 0.85 (0.54-1.36) |
| 2.0-2.9 ng/ml | 2615 | 1.11 (0.68-1.81) | 1.01 (0.61-1.67) | 1151 | 0.74 (0.46-1.20) | 0.76 (0.47-1.25) |
| 3.0-3.9 ng/ml | 1688 | 1.25 (0.74-2.12) | 1.11 (0.64-1.92) | 790 | 0.78 (0.46-1.32) | 0.80 (0.47-1.37) |
| 4.0-4.9 ng/ml | 1134 | 1.32 (0.73-2.37) | 1.07 (0.57-1.98) | 548 | 0.80 (0.45-1.43) | 0.76 (0.41-1.40) |
| 5.0-5.9 ng/ml | 706 | 1.12 (0.54-2.34) | 0.95 (0.45-2.03) | 339 | 0.69 (0.33-1.42) | 0.68 (0.32-1.43) |
| 6.0-6.9 ng/ml | 405 | 1.52 (0.67-3.43) | 1.24 (0.54-2.86) | 188 | 0.96 (0.43-2.15) | 0.90 (0.40-2.05) |
| 7.0-7.9 ng/ml | 306 | 2.59 (1.24-5.38) | 2.02 (0.94-4.34) | 145 | 1.60 (0.78-3.29) | 1.42 (0.67-3.00) |
| 8.0-9.9 ng/ml | 308 | 2.57 (1.24-6.35) | 2.06 (0.96-4.41) | 146 | 1.59 (0.77-3.27) | 1.47 (0.70-3.09) |
| 10-max ng/ml | 361 | 3.66 (1.99-6.70) | 2.69 (1.35-5.36) | 147 | 2.63 (1.46-4.75) | 2.18 (1.12-4.23) |

Adjusted for female age, BMI, parity, primary infertility, ectopic pregnancy history, ovulatory dysfunction, tubal factor.
